# Investigating the Role of the Eurasian Badger (*Meles meles*) in the Nationwide Distribution of the Western European Hedgehog (*Erinaceus europaeus*) in England

**DOI:** 10.3390/ani9100759

**Published:** 2019-10-09

**Authors:** Anouschka R. Hof, Andrew M. Allen, Paul W. Bright

**Affiliations:** 1 Resource Ecology Group, Wageningen University, 6708 PB Wageningen, The Netherlands; 2 Department of Wildlife, Fish, and Environmental Studies, Swedish University of Agricultural Sciences, SE-907 36 Umeå, Sweden; 3 School of Biological Sciences, Royal Holloway, University of London, Egham TW20 0EX, UK; drp.w.bright@gmail.com; 4 Department of Animal Ecology & Physiology, Radboud University, 6500 GL Nijmegen, The Netherlands; Andrew.Allen@science.ru.nl

**Keywords:** citizen science, conservation, displacement, predator-prey interaction, spatial use

## Abstract

**Simple Summary:**

The hedgehog is a species known to many in society. What is perhaps less known, is that the hedgehog has been declining across large parts of Europe, including the United Kingdom. Effective hedgehog conservation requires a sound understanding of the causes of the decline. A potential cause is the badger, whose population has been recovering in recent years. The badger is an intraguild predator of the hedgehog, meaning that not only do the two species share the same food, like snails and earthworms, but badgers also predate on hedgehogs. Our study investigates how the presence of hedgehogs is related to the presence of badgers, along with other landscape features. Using information from two nationwide citizen science surveys, we first determine where both species can be found and then identify which factors best explain hedgehog presence. We found that the badger was indeed important, and hedgehogs were less likely to be found in areas where badgers were likely to be found. Interestingly, hedgehogs were also likely to be found in arable land, a habitat not directly thought to be favourable for hedgehogs. Badgers may, therefore, be an important consideration when designing hedgehog conservation plans, and further research of these impacts is needed.

**Abstract:**

Biodiversity is declining globally, which calls for effective conservation measures. It is, therefore, important to investigate the drivers behind species presence at large spatial scales. The Western European hedgehog (*Erinaceus europaeus*) is one of the species facing declines in parts of its range. Yet, drivers of Western European hedgehog distribution at large spatial scales remain largely unknown. At local scales, the Eurasian badger (*Meles meles*), an intraguild predator of the Western European hedgehog, can affect both the abundance and the distribution of the latter. However, the Western European hedgehog and the Eurasian badger have shown to be able to co-exist at a landscape scale. We investigated whether the Eurasian badger may play a role in the likelihood of the presence of the Western European hedgehog throughout England by using two nationwide citizen science surveys. Although habitat-related factors explained more variation in the likelihood of Western European hedgehog presence, our results suggest that Eurasian badger presence negatively impacts the likelihood of Western European hedgehog presence. Intraguild predation may, therefore, be influencing the nationwide distribution of hedgehogs in England, and further research is needed about how changes in badger densities and intensifying agricultural practices that remove shelters like hedgerows may influence hedgehog presence.

## 1. Introduction

In a time of ongoing anthropogenic pressures on nature, a growing number of species throughout the world are facing population declines [1]. One of these species is the Western European hedgehog (*Erinaceus europaeus*). Albeit being classified as least concern on the International Union for Conservation of Nature (IUCN) red list of threatened species, hedgehog numbers appear to have fallen in several countries in Europe in the last couple of decades, such as in Belgium and the Netherlands [2,3], Sweden [4], and in the United Kingdom [5,6,7,8,9,10]. Reasons behind this decline are, however, currently unclear and several potential causes have been suggested. The network of roads throughout Europe has been increasing extensively over the past few decades [11], which may play a large role since hedgehogs often fall victim to traffic [2,3,12] and large roads may act as barriers [13]. Increasing demands for housing development may coincide with the loss of greenspaces in built-up areas that offer (sub)urban-dwelling species like hedgehog habitat and refuge [14,15]. Agricultural intensification may lead to decreasing habitat suitability and also reduce resource availability of macro-invertebrates [16,17,18]. Furthermore, agricultural intensification may lead to reduced landscape complexity, for example, the removal of hedgerows and coppices [19], which provide hedgehogs with shelter from predators and nesting habitats [14,20]. In addition, Eurasian badgers (*Meles meles*) may influence hedgehog populations at local scales [14,21], but largescale studies investigating the impact of such factors are currently lacking.

The role of the Eurasian badger as a potential driver for the nationwide decline of hedgehogs requires further research as it has been suggested as a potential driving factor of declining hedgehog populations following increased predation pressure [14]. Hedgehogs and badgers are in a guild of generalist predators of macro-invertebrate prey [20,22], but badgers may also be an intraguild predator of hedgehogs. Although many studies have not reported hedgehog remains in badger diet analyses [23,24,25], and incidences of badgers preying on hedgehogs are not thought to be common, there are several studies throughout Europe that do report hedgehog remains in the faeces or stomachs of badgers. Hedgehog occurrence in the diet of badgers varied from as much as four hedgehog remains in the stomach of one single adult badger found in England [26], to an 11.2% occurrence in badger scats in one of three study sites in Poland [27], 2.9% occurrence in badgers scats in Italy [28], and an unknown percentage of occurrence in an extensive review of dietary studies in the former Soviet Union [29].

Predation, both intraguild and interguild, is known to be important in shaping the local dynamics of predator–prey communities [30,31]. In fact, several fine-scale studies show that badgers can have large negative effects on local hedgehog populations [32,33,34,35]. Experimental evidence of the Randomised Badger Culling Trial in 100 km^2^ large trial areas in England, set up to assess impacts of culling on the incidence of bovine tuberculosis, provided evidence that hedgehog numbers more than doubled over a five year period in areas with preferred habitat [36]. Furthermore, badgers appear to drive small scale movement patterns of hedgehogs [32,37,38]. Doncaster [21] showed, using an experimental setup in which a low-density hedgehog population was artificially increased, and a high-density population was artificially decreased, that predation by badgers can affect both hedgehog abundance and their distribution at a local scale. However, both species have shown to be able to coexist on a landscape scale in the recent past [34]. Yet, the number of badgers has been increasing in the past couple of decades; Judge et al. [39] estimated that there had been an 88% increase in badger numbers across England and Wales from the 1985–1988 to the 2011–2013 survey periods. The balance of co-existence between badgers and hedgehogs may, therefore, have been tipped. A study by Williams et al. [9], which investigated correlates of hedgehog presence in rural England and Wales using footprint tracking tunnels at 261 sites, found a strong negative relationship between hedgehog occupancy and badger sett density, but simultaneously that hedgehogs were absent from 71% of surveyed sites that had no badger setts. Consequently, factors driving the distribution of hedgehogs at large spatial scales, i.e., throughout the United Kingdom, remain uncertain. There is no indication that other potential predators of hedgehogs in the United Kingdom, such as the red fox (*Vulpes vulpes*), can regulate hedgehog presence [15,21,34].

As the abundance and distribution of species has a propensity to be linked, where widespread species tend to be more abundant, a thorough understanding of the drivers behind large scale, e.g., nationwide scale, distributions of species is highly valuable in complementing knowledge obtained from small scale studies and designing and implementing conservation measures at large scales [40]. The objective of our study was, therefore, to investigate the factors driving the distribution of hedgehogs on a large scale—the whole of England—and to investigate whether the presence of badgers and other landscape features explain some variation in the nationwide distribution of hedgehogs. We used two nationwide citizen science surveys and land cover data to investigate the impact of badgers and landscape features on the distribution of hedgehogs, which may provide valuable knowledge for the nationwide conservation of hedgehogs.

## 2. Materials and Methods

The current distribution of hedgehogs in England was obtained by a nationwide citizen science survey called ‘HogWatch’. The survey was mainly designed by the British Hedgehog Preservation Society (BHPS, https://www.britishhedgehogs.org.uk/) and the People’s Trust for Endangered Species (PTES, https://ptes.org/) in conjunction with Royal Holloway, University of London. It was both post and web based. Publicity was sought by means of (local) media, personal communication, and using existing member databases of the BHPS and the PTES. The survey, ‘HogWatch’ is no longer active but PTES collect annual hedgehog records through surveys like Mammals on Roads (https://ptes.org/get-involved/surveys/road/) and ‘Living with Mammals’ (https://ptes.org/get-involved/surveys/garden/living-with-mammals). PTES and BHPS also collect records of hedgehogs through their Big Hedgehog Map (https://bighedgehogmap.org/). A total of 19,184 people provided 25,911 presence and absence reports of hedgehogs that were distributed throughout England. The hedgehog distribution was based on 25,911 grid-referenced sightings and lack of sightings of living hedgehogs from 2005 (when the survey started) and 2006. Data on badger presence in England were derived from another survey called ‘Living with Mammals’ (https://ptes.org/get-involved/surveys/garden/living-with-mammals), which is open to every interested member of the public and was originally designed by the Mammals Trust UK in conjunction with Royal Holloway, University of London. Surveyors recorded badger presence throughout 13 consecutive weeks from the start of April, and they stated the approximate observation length during dawn, daytime, dusk, and night-time. The badger distribution was based on 2,703 sightings of living badgers recorded in 247 grid-referenced sites and the lack of sightings of living badgers recorded in 1464 sites throughout England in the years 2003–2006. We included badger data from 2003 (the first year the survey was held) and 2004 in our analyses as well since it allowed us to include a greater sample size and have a smaller discrepancy with the substantially larger ‘HogWatch’ dataset. In addition, we felt that the distribution of badgers would not rapidly change to a large extent from the years 2003–2004 to 2005–2006. Although respondents were asked to state the date, time, and approximate length of the observation, to get an estimate of effort, this effort was not considered, as such data were not collected for the ‘HogWatch’ dataset. We assumed that the relative density of hedgehogs/badgers is proportional to the actual density and that the rate of proportionality is constant [41].

**Figure 1:**
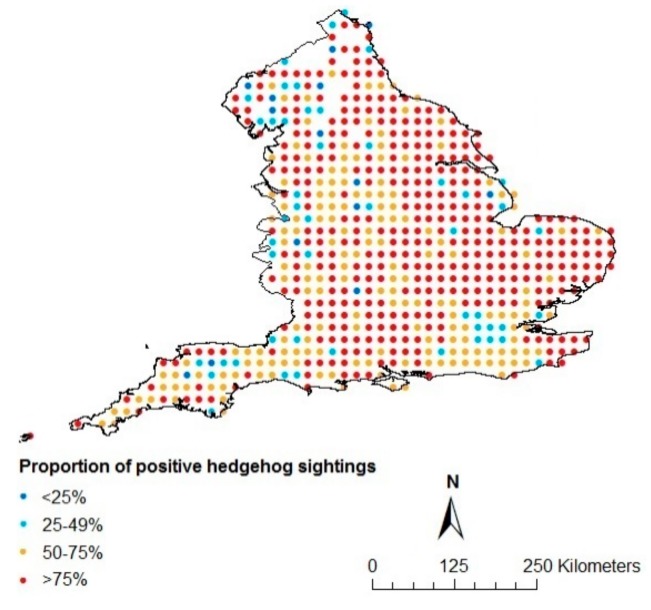
Proportion of positive hedgehog sightings according to the ‘HogWatch’ survey of 2005–2006.

**Figure 2:**
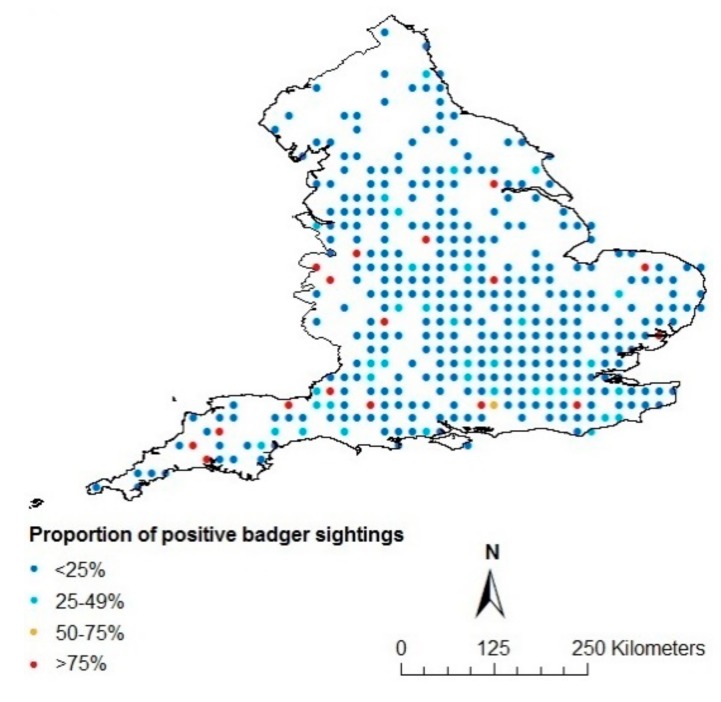
Proportion of positive badger sightings according to the ‘Living with Mammals’ survey of 2003–2006.

**Figure 3:**
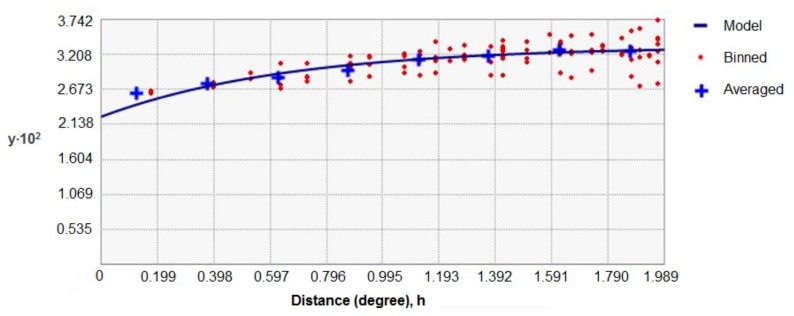
The exponential semivariogram model with 8 lags used for kriging the hedgehog data.

**Figure 4:**
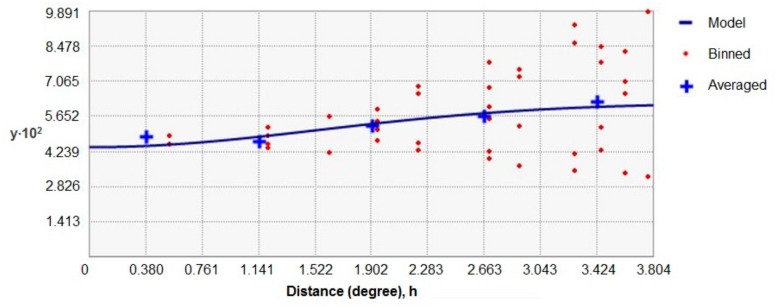
The Gaussian semivariogram model with 5 lags used for kriging the badger data.

As the surveys used to obtain data about hedgehog and badger presence/absence did not overlap with each other at a fine scale, since they were not especially designed for this study, we used ordinary kriging [42] to estimate the likelihood of the presence of hedgehogs and badgers throughout England at a 10 km^2^ scale using ArcMap 10.5 (Environmental Systems Research Institute [ESRI], Redlands, CA, USA). We chose a 10 km^2^ scale to obtain a reasonable number of species observations per cell, while still retaining some level of detail. Kriging is a geostatistical interpolation method that is based on linear regressions, and produces maps from irregular spatial data to visualize suggested trends and spatial differences in the likelihood of presence. The method is frequently used in spatial prediction applications in ecology [42,43,44,45] to, amongst others, predict species numbers in regions where data are not available [46,47,48,49]. Interpretations based on results derived from kriging must, however, be made with care. The proportion of respondents to the surveys that reported seeing hedgehogs/badgers per 10 km^2^ were used as input for the kriging (Figure 1 and Figure 2). To account for spatial autocorrelation, a semivariogram, which is a function describing the degree of spatial dependence of the data and characterizes the spatial continuity or roughness of data sets, was integrated in the kriging method, as is recommended [50,51]. An exponential model was used with 8 lags for the hedgehog (Figure 3) and a Gaussian model with 5 lags for the badger (Figure 4), and nuggets were enabled. The type of model and the amount of lags were chosen based on the smallest standard errors showing the uncertainty related to the predicted values. Maps, showing the likelihood of the presence of badgers and hedgehogs in each 10 km^2^ grid-cell in England were produced in ArcMap10.5 (Environmental Systems Research Institute [ESRI], Redlands, CA, USA). The methodology allowed us to obtain maps depicting differences in the likelihood of the presence of the hedgehog and of the badger at a 10 km^2^ scale for the entirety of England. Since the red fox does not appear to be able to regulate hedgehog presence [15,21,34], we did not include the likelihood of the presence of red foxes in our study.

Landscape features that may also be important in shaping the distribution of hedgehogs throughout England were obtained from various sources. In addition to the likelihood of the presence of badgers as described above, these variables included habitat-related variables and the density of built-up areas, which were obtained from the Land Cover Map 2000 (https://www.ceh.ac.uk/services/land-cover-map-2000) and the Land Cover Map 2007 (https://www.ceh.ac.uk/services/land-cover-map-2007) from the Centre for Ecology & Hydrology (https://www.ceh.ac.uk/), data on the human footprint obtained from the Socioeconomic Data and Applications Center (SEDAC, https://sedac.ciesin.columbia.edu/data/set/wildareas-v2-human-footprint-geographic) [52], and soil-related variables, which were obtained from the National Soil Resource Institute (NSRI, https://www.cranfield.ac.uk/centres/soil-and-agrifood-institute/research-groups/national-soil-resources-institute). The land cover data from the land cover maps are classifications of spectral data recorded by satellites and refined using external datasets. For more information about the methodology used to create the land cover maps, please refer to the final reports of the surveys [53,54]. Data were unfortunately not available for the exact timeframe when the hedgehog and badger data were collected (2003–2006), but only for 2000 and 2007. However, changes in landscape features between both timeframes were expected to be minor. We considered both the target (more detailed classification) and aggregate (less detailed classification) classes. The data were available at a scale of 1 km^2^ and were converted to 10 km^2^ by taking the mean and median of the values (if continuous) for each 10 km^2^. In the case of soil data, presence (1) or absence (0) of a soil type was used. All variables used are shown in Table 1.

We used generalised linear modelling (GLM) to determine how the likelihood of hedgehog presence (non-kriged values, i.e., the proportion of respondents reporting a hedgehog sighting) was related to the (kriged) likelihood of badger presence and landscape features (Table 1). All explanatory variables were simultaneously tested for correlation and visualised using a correlation matrix. Any highly correlated (r > 0.7, [55]) variables were either excluded from the model or were not included simultaneously in the same model. We also checked the collinearity of explanatory variables in the final models using variation inflation factors (VIFs), where a VIF > 4 indicates collinearity and a VIF > 10 indicates severe collinearity [56]. An initial model was built that included all explanatory variables, and the most parsimonious model was obtained by stepwise backward selection, removing the least significant variable in each step. The Akaike Information Criterion (AIC) was compared to also determine whether the model-fit significantly changed (i.e., ∆AIC > 2; [57]). The final model would thus contain significant variables only.

We also considered a multi-model selection approach that compares all possible model combinations. Due to the large number of potential explanatory variables, we aimed to identify the most parsimonious model to avoid model overfitting. Models were ranked according to the Bayesian Information Criterion (BIC), which has a larger penalty for additional parameters compared with AIC and AICc [57]. Models with ∆BIC < 2 have substantial support, whilst models with a ∆BIC > 4 have considerably less support [57]. Therefore, we considered top models to be those within ∆BIC < 2. However, when considering variables that may be important for explaining the likelihood of hedgehog presence, we considered all variables included in models with ∆BIC < 4. The analysis was performed using the dredge function in the MuMIn package in R 3.5.1 [58,59]. We used hierarchical partitioning, which calculates the explained variation (R^2^) for all combinations of the supplied variables in a regression hierarchy, to identify the individual contribution of each variable to the explained variation [60,61].

## 3. Results

**Figure 5:**
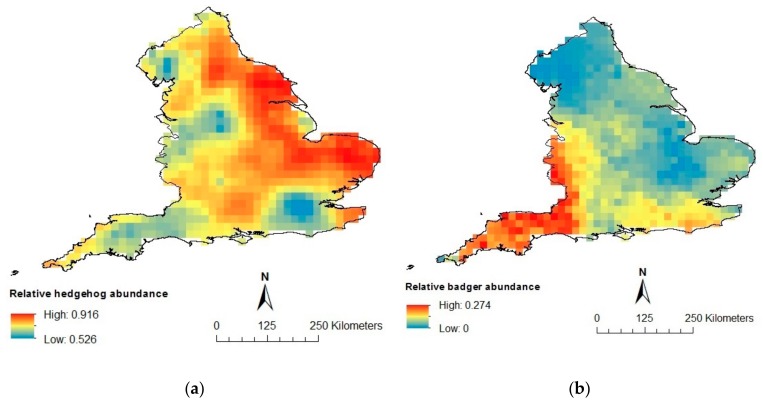
Maps showing an index (low: 0, high: 1) of the likelihood of the presence of (**a**) hedgehogs and (**b**) badgers throughout England.

We found that the likelihood of badger presence was highest in the southern and western parts of England (Figure 5). Simultaneously, there was a division in the likelihood of the presence of hedgehogs between the eastern and the southern and western parts of England, with a higher probability of hedgehog presence in eastern England.

**Figure 6:**
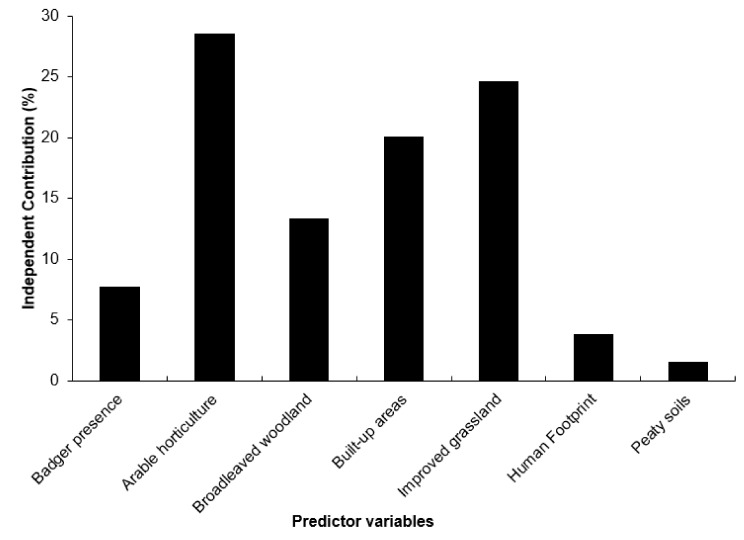
Results of hierarchical partitioning showing the individual contribution of each variable towards the total explained variation (R^2^) of the model (R^2^ = 0.258).

We present our results using median values for the land cover maps of 2007, but results were comparable when using either median or mean for both 2000 and 2007 land cover maps (Appendix A, Tables S1–S3). Both stepwise backwards selection and the multi-model selection approach included the same variables in the top model, meaning these variables explained most of the variation in hedgehog presence and that all variables were significant (Table 2 and Table 3). Arable land had a positive relationship with hedgehog presence, whilst broadleaved woodland, improved grassland, built-up areas, and badger presence were negatively associated with hedgehog presence (Table 2). The multi-model selection confirmed the importance of badgers in explaining hedgehog presence, which was included in the top two models (Table 3). Human footprint and peaty soils were also amongst the top models with a ∆BIC < 4. Hierarchical partitioning of the seven variables included within models of ∆BIC < 4 (Table 3) indicated that arable land, built-up area, and improved grasslands explained the most variation, each with an independent contribution of more than 20% towards the explained variation (Total R^2^ = 0.258; Figure 6). Amongst the other variables, broadleaved woodlands were the most important (13.4%) followed by badgers (7.8%), human footprint (4.9%), and peaty soils (1.6%; Figure 6).

## 4. Discussion

Our results show that the nationwide distribution of hedgehogs was negatively related to that of its intraguild-predator, the badger. Arable land and the density of improved grassland were the strongest predictors of the likelihood of the presence of hedgehogs throughout England, followed by the amount of built-up area. The likelihood of hedgehog presence was negatively correlated with the density of improved grassland and the amount of built-up area but positively correlated with arable land. The likelihood of hedgehog presence was, thus, higher in the arable dominated landscapes of eastern England than in other parts of England, which agrees with previous findings [15,62]. Although badger presence was not the most important variable for explaining nationwide hedgehog presence, badger presence was, nonetheless, amongst the most important variables. When one considers that the likelihood of hedgehog presence was higher in areas that are supposedly less accommodating to their needs, i.e., areas dominated by arable land [20,63], it begs the question whether the lack of badger presence may lead to proportionally higher hedgehog numbers in less accommodating areas. Badgers were more likely to be present in the south-west, which is largely in agreement with previous findings [38,64] and correlates with the lower likelihood of the presence of hedgehogs in these regions. One of the reasons that badgers are likely to be present at higher densities in the south than in the east of England might be partly due to the higher density of broadleaved woodland and hillier and undulating habitat, environmental features favoured by badgers [65,66].

The negative relationship between hedgehog presence and the density of improved grassland and built-up areas was expected based on previous studies [20,63,67], but the positive relationship with arable land was unexpected given that radio-tracking studies indicate that hedgehogs often avoid these habitats [20,63]. The positive relationship with arable land was also contrary to general expectations based upon studies that investigated habitat preferences of hedgehogs at home range and landscape scales [20,68,69,70]. Which aspects of arable land were particularly related to hedgehog presence remains unclear due to the limited detail of the land cover data. Small scale, less intensively farmed areas with, for example, hedgerows, may be more attractive to hedgehogs than large scale intensively farmed areas with single crops. Unfortunately, detailed data about land-use intensity is lacking, and although we included human footprint, this also had a positive relationship with hedgehog presence, which is likely due to hedgehog’s association with agricultural landscapes. Furthermore, detailed data about the presence of hedgerows throughout the country were not available and may further clarify the trends we observed. Further research at finer spatial studies may clarify which factors drive the positive relationship between arable land and hedgehog presence. A study by Hof et al. [37], however, indicates that the small-scale utilization of arable land can especially depend on the presence of badgers.

It is not known whether the geographical distribution of the hedgehog has changed in the recent past, or whether the likelihood of the presence of the hedgehog has been higher in the arable dominated areas in England for a long time already. However, whilst the numbers of hedgehogs seem to be falling in spite of protection [5,6,7,8,9,10], the number of badgers has been steadily increasing in recent decades following their protection [39,71]. This might have tipped the balance of co-existence between badgers and hedgehogs in favour of badgers and initiated a change in the likelihood of the presence of the hedgehog in England. That this may indeed be so is corroborated by research from Micol et al. [34], who predicted that apart from some isolated pockets, hedgehogs will be absent from most sites in the United Kingdom with badger sett densities above 0.23 per km^2^. A recent study by Judge et al. [39] actually estimated that current badger sett densities were 0.49 per km^2^ in England between 2011 and 2013, thus substantially larger than the 0.23 limit suggested by Micol et al. [34]. However, it cannot be ruled out that differences in survey protocols (partly) explain this difference in sett density as well. Furthermore, badger numbers are poorly predicted by sett characteristics [72].

Another line of explanation of the geographical distribution pattern of hedgehogs in England is offered by the hypothesis that the presence of an intraguild-prey species is partly dependent on the availability of alternative food sources for the intraguild-predator [73]. Generally, most studies have found that vertebrate prey, and especially mammalian prey, make up a small component of the badger’s diet, which is generally dominated by cereals, fruits, and invertebrates [23,74,75]. Nonetheless, prey composition varies spatially, and our results may, therefore, suggest that western England currently offers a low abundance of alternative prey sources (e.g., macro-invertebrates). Low availability of prey sources would, thus, not only increase competition between badgers and hedgehogs but may also increase predation rates of the badger on hedgehogs. The low abundance of prey sources may possibly be an effect of intensified farming [76]. Detailed data on the England wide abundance of macro-invertebrates were, however, not available to include in this study. Yet, overwhelming evidence indicates that invertebrates are declining in agricultural landscapes across the globe [77,78], it can thus not be ruled out that declining invertebrate numbers did not only play a role in declining hedgehog numbers, but also in shaping their distribution. Other factors that may need investigation include possible impacts of disease, pesticides, and of potential predators other than the badger, such as feral/stray dogs and foxes. There are a large range of diseases and parasites that can negatively affect hedgehogs [20], but it thus far remains uncertain if they play a significant role in population declines [79]. We found no published evidence of disease transmission between badgers and hedgehogs. Disease transmission between the two species may be low, considering the fact that a prevalent disease in badgers, bovine tuberculosis, was not prevalent in wild hedgehogs in the United Kingdom [80]. It is known that pesticides can accumulate in hedgehogs [81]; the extent of negative effects and impacts on population declines, however, remain uncertain. There is currently no evidence that feral/stray dogs and foxes are able to regulate hedgehogs [15,21,34], they are, however, able to occasionally kill or inflict injury upon hedgehogs [20]. The potential impacts of feral/stray dogs and foxes are therefore expected to be more pronounced in small geographic scales rather than at large geographic scales.

Our study relies upon hedgehog sightings reported by citizen scientists through an initiative called ‘HogWatch’. A benefit of using these is that we attained nationwide coverage with responses received from almost every 10 km^2^ grid cell in England. Furthermore, nearly 26,000 responses were received from approximately 20,000 respondents. This attains a much larger spatial scale compared to field-based studies, such as the one recently conducted by Williams et al. [9]. However, citizen science survey data also have associated challenges that should be taken into account. For example, we made the assumption that a higher proportion of reports on hedgehog/badger presence per 10 km^2^ grid-cell was positively related to the likelihood of presence. This assumption needs to be made with some care because of recordings of possible false absences and dissimilarity in visibility caused by differences in human habitation and environmental features. Furthermore, both species are nocturnal with higher chances of activity at night when people are less likely to see them. However, the ‘HogWatch’ survey was specifically designed for hedgehogs, and the respondents to the ‘Living with Mammals’ survey also surveyed during nightly hours. Considering the large amount of data collected and the spread of data throughout England in both surveys, it was assumed that the nocturnal activity pattern of both species had little effect on the spatial distribution patterns of sightings. Furthermore, both species are unmistakable with other mammals, so errors caused by misidentification are thought to be negligible. In addition, with regards to the survey to obtain data on hedgehogs, there is a possibility that possible trends may have emerged because of geographic differences in the eagerness of people to respond to surveys, although the participants of the survey were spread throughout England (Figure 1). Our analysis incorporates some of these challenges by, for example, only analysing the data at the 10 km^2^ scale [82] and correcting for spatial-autocorrelation in the kriging process to improve confidence about the conclusions drawn from our study. All of these potential biases and challenges involved with citizen science data should not be underestimated and results coming from studies using citizen science data should be interpreted with caution. However, there are several benefits to such data as well, not least the potential to use data often collected at large geographic scales and on private lands, which can be difficult to obtain using more traditional survey approaches [83].

## 5. Conclusions

Whilst it was already known that badgers could regulate hedgehogs on smaller scales [33,34,35,36,37], the role badgers play at the nationwide scale was less clear. The negative relationship between the likelihood of hedgehog and badger presence observed in our study suggests that badgers may at least partly explain the variation in the presence of hedgehogs throughout England. These findings mirror findings by Williams et al. [9] who surveyed 261 sites in rural England and Wales using footprint tunnels to determine site occupancy by hedgehogs and badger sett presence as a proxy for the relative density of badgers. These combined results stress that it is imperative for the conservation of species to fully understand community interactions. Neglecting important community interactions, such as predation, may prevent us from recognising drivers of dynamics in species abundance and distribution, which may consequently lead to inappropriate conservation measures. In the case of the hedgehog, although nationwide hedgehog presence was negatively correlated with that of badgers, other, habitat-related, factors were stronger predictors. The potential role of declining macro-invertebrate abundance, therefore, needs further investigation.

## Figures and Tables

**Table 1:** Explanation of the response variables used (LCM: Land Cover Map 2000 and 2007, NSRI: National Soil Resource Institute, LWM: Living with Mammals survey, SEDAC: Socioeconomic Data and Applications Center [see text for explanation]) to study their impact on the relative hedgehog abundance throughout England in 2005–2006. Some land classes from the LCM were not included because they were extremely rare (absent from >90% of grid cells) or were not suitable, such as “Mountain, heath, bog”, “Saltwater”, “Freshwater”, and “Coastal”.

Variable	Explanation	Source
Arable land	Proportion of arable and horticultural area	LCM
Badger presence	Index of relative badger abundance 2003–2006	LWM
Broadleaf woodland	Proportion of broadleaved woodland	LCM
Built-up	Proportion of built-up area (includes target classes of urban and sub-urban)	LCM
Coniferous woodland	Proportion of coniferous woodland	LCM
Human footprint	Human Influence Index normalized by biome and realm	SEDAC
Improved grassland	Proportion of improved grassland	LCM
Semi-natural grassland	Proportion of semi-natural grassland (includes target classes of rough, neutral, calcareous, acid grassland, and fen, marsh and swamp)	LCM
Soil type	The soil types of England 1: soils with a clay texture, 2: soils with a peaty texture, 3: soils with a sandy texture, 4: soils with a loamy texture and rich in lime, 5: soils with a loamy texture and a low fertility, 6: soils with a loamy texture and a moderate to high fertility	NSRI

**Table 2:** Model results of generalised linear modelling (GLM) explaining the likelihood of the presence of hedgehogs. Significant variables were determined using backwards stepwise selection. SE = standard error and *p* = *p*-value. The independent contribution of each variable towards the explained variation (R^2^; total = 0.242) was measured using hierarchical partitioning and VIF is the variance inflation factor.

Variable	Coefficient	SE	*p*	R^2^	VIF
Intercept	0.834	0.029	<0.001	-	-
Badger presence	−0.078	0.030	0.010	0.021	1.047
Arable land	0.090	0.036	0.012	0.073	1.671
Built-up	−0.238	0.056	<0.001	0.045	1.367
Improved grassland	−0.245	0.053	<0.001	0.068	1.422
Broadleaved woodland	−0.419	0.141	0.003	0.035	1.151

**Table 3:** Top-performing models with ∆BIC < 4 from a multi-model selection consisting of all possible explanatory variables. Shaded areas indicate that the variable was included in the model. All model variables had VIFs < 2. LogL is the log-likelihood, Arable is Arable Horticulture, Badgers is the likelihood of badger presence, BLwood is broadleaved woodland, ImprG is improved grasslands, HFI is human footprint index, and Peat is soils with a peaty texture.

∆BIC	LogL	Arable	Badgers	BLwood	Built-Up	ImprGr	HFI	Peat
0.00 ^1^	223.05							
0.46	219.85							
0.76	219.70							
0.87	216.68							
1.20	225.42							
1.81	222.15							
2.46	224.79							
2.96	218.60							
2.99	221.56							
3.22	221.44							
3.40	218.39							
3.84	221.13							
3.98	227.00							

^1^ BIC = −404.54.

## Supplementary Materials

The following are available online at https://www.mdpi.com/2076-2615/9/10/759/s1, Table S1: results using mean values for the land cover maps of 2007, Table S2: results using median values for the land cover maps of 2000, Table S3: and results using mean values for the land cover maps of 2000.

## Appendix A Results Comparison Among Landcover Maps

The data collection of our study fell between two periods during which landcover maps were produced for the UK. Consequently, we compared our results from the landcover maps for both 2000 and 2007. Furthermore, landcover metrics can be calculated in different ways, for example taking the median percentage cover in a 10km^2^ grid cell or taking a mean. We present the results from median landcover in 2007 and here in this Appendix we include mean landcover for 2007, and also median and mean landcover for 2000.

The most important variables remained the same in all analyses, namely arable and horticulture, improved grassland, built-up areas, broad-leaved woodlands and badger presence (Tables S1–S3). Using a mean human footprint index explained more variation than a median human footprint index (Tables S1–S3). When using mean landcover data for 2007, semi-natural grassland and loamy soils were also included within models of ∆BIC < 4, both of which had a positive relationship with hedgehog presence but had a low independent contribution towards the variation explained (Table S1).

The landcover map of 2000 included additional target classes for arable land, namely arable cereals, arable horticulture and arable non-rotational, which were not available in the 2007 data. Therefore, we determined whether these three separate variables performed better in explaining hedgehog presence than the aggregated landcover variables of arable and horticulture (which combined all three sub-classes). It was evident that arable non-rotational had a negative relationship with hedgehog presence, but this was not significant and was not included within any of the models with ∆BIC < 4. The sub-class of arable cereal and arable horticulture were included, and both had a positive relationship with the likelihood of hedgehog presence, but only the sub-class of arable and horticulture was included in the top model. Despite the separate contributions of the arable sub-classes, the aggregated variable for arable and horticulture (includes all three arable types) provided a better model fit (BIC-400.93) than the target class arable and horticulture alone (BIC -401.96).

Using a median landcover value per grid cell, the most important variables remained the same as the main presented results (Table S2). An exception is that neutral grassland, coniferous forest and loamy soils were included in models with ∆BIC < 4 and had a positive relationship with the likelihood of hedgehog presence, but these had a minor overall contribution towards the explained variation (Table S2).

The results of the models using a mean landcover value (Table S3), instead of a median (Table S2), for the landcover map 2000 were very similar, and also to the main results presented in the article. The only notable exception is that the variable improved grassland was excluded from the top models (Table S3). The effect of improved grassland remained negative however the contribution towards model performance was reduced. This is somewhat evident in the results using a median value since improved grassland explained less variance and was not significant (Table S2) in comparison with the 2007 values (Table S1). This change may indicate that changes to grassland habitats, most notably intensifying use, may have increased in the period between 2000 and 2007. The inclusion of the variable neutral grasslands in top performing models using landcover maps from 2000 (Tables S2 and S3), and exclusion of neutral grasslands in top performing models using landcover maps from 2007 (Tables S1 and S3) may support this.
