# Supplementary material for: Investigating the Role of the Eurasian Badger (Meles meles) in the Nationwide Distribution of the Western European Hedgehog (Erinaceus europaeus) in England
Source: Animals (Basel). 2019 Oct 9;9(10):759. doi: 10.3390/ani9100759 (PMC6826801; doi:10.3390/ani9100759)
Supplement: Supplementary file 1 [file animals-09-00759-s001.pdf]

Investigating the Role of the Eurasian Badger (*Meles meles*) in the Nationwide Distribution of the Western European Hedgehog (*Erinaceus europaeus*) in England

Anouschka R. Hof, Andrew M. Allen [4] and Paul W. Bright

Table S1. Model analyses using mean values from landcover maps of 2007, showing coefficients and significance of all variables included within ΔBIC < 4 of the top model. The variables included in the top model are shown with an asterix (*). The independent contribution of each variable towards the explained variation (R^2) was estimated using hierarchical partitioning, and the percent contribution towards the total explained variation ($R^2 = 0.282$) is shown by I (%). VIF is the variance inflation factor and SE is the standard error.

Variable	Coefficient	SE	*p*	R^2	I (%)	VIF
Intercept	0.623	0.070	<0.001	-	-	-
Badgers *	−0.080	0.030	0.007	0.021	7.32	1.056
Arable & horticulture *	0.144	0.048	0.002	0.073	25.9	2.365
Broadleaf woodland *	−0.361	0.131	0.006	0.037	13.2	1.188
Built-up *	−0.474	0.088	<0.001	0.054	19.5	3.251
Improved grassland *	−0.166	0.069	0.016	0.057	20.3	1.876
Human Footprint *	0.368	0.089	<0.001	0.024	8.5	3.559
Semi-natural grassland	0.239	0.154	0.122	0.005	1.7	1.522
Peaty soils	−0.030	0.020	0.131	0.004	1.3	1.045
Loamy soils	0.042	0.030	0.166	0.007	2.4	1.044

Table S2. Model analyses using median values from landcover maps of 2000, showing coefficients and significance of all variables included within ΔBIC < 4 of the top model. The variables included in the top model are shown with an asterix (*). The independent contribution of each variable towards the explained variation (R^2) was estimated using hierarchical partitioning, and the percent contribution towards the total explained variation ($R^2 = 0.265$) is shown by I (%). VIF is the variance inflation factor and SE is the standard error.

Variable	Coefficient	SE	*p*	R^2	I (%)	VIF
Intercept	0.649	0.053	<0.001	-	-	-
Badgers *	−0.077	0.030	0.012	0.019	7.47	1.070
Arable horticulture *	0.164	0.042	<0.001	0.073	27.7	2.168
Broadleaf woodland *	−0.405	0.136	0.003	0.038	14.2	1.162
Built-up *	−0.373	0.071	<0.001	0.056	21.2	2.187
Improved grassland	−0.100	0.062	0.108	0.042	15.7	1.982
Human Footprint *	0.235	0.067	<0.001	0.015	6.0	2.529
Coniferous woodland	0.715	0.455	0.117	0.002	0.9	1.123
Neutral grassland *	0.657	0.234	0.005	0.010	4.0	1.235
Loamy soils	0.047	0.031	0.123	0.007	2.7	1.041

Table S3. Model analyses using mean values from landcover maps of 2000, showing coefficients and significance of all variables included within ΔBIC < 4 of the top model. The variables included in the top model are shown with an asterix (*). The independent contribution of each variable towards the explained variation (R^2) was estimated using hierarchical partitioning, and the percent contribution towards the total explained variation ($R^2 = 0.274$) is shown by I (%). VIF is the variance inflation factor and SE is the standard error.

Variable	Coefficient	SE	*p*	R^2	I (%)	VIF
Intercept	0.499	0.045	<0.001	-	-	-
Badgers *	−0.085	0.029	0.004	0.023	8.5	1.036
Arable horticulture *	0.235	0.041	<0.001	0.096	35.1	1.585
Broadleaf woodland	−0.283	0.134	0.034	0.039	14.3	1.160
Built-up *	−0.520	0.091	<0.001	0.056	20.3	3.267
Human Footprint *	0.462	0.081	<0.001	0.036	13.1	2.973
Neutral grassland *	0.705	0.182	<0.001	0.015	5.4	1.288
Loamy soils	0.053	0.030	0.080	0.008	3.1	1.024
